# Supplementary material for: Host-specific gene expression as a tool for introduction success in Naupactus parthenogenetic weevils
Source: PLoS One. 2021 Jul 30;16(7):e0248202. doi: 10.1371/journal.pone.0248202 (PMC8323892; doi:10.1371/journal.pone.0248202)
Supplement: S1 Fig — (i) host-specific expression between weevils feeding on a) Legumes vs. Other (for N. cervinus and N. leucoloma), b) Legumes vs. Citrus, c) Conventional vs Organic orange hosts and d) Switch vs Maintain. (ii) contrasts between expression levels while feeding on host plants from the same family Citrus vs. Citrus (Rutaceae:Citrinae), Legume vs Legume (Fabaceae) and Other vs Other (Asteraceae). Shades of red indicate upregulation in Group 1 while shades of blue indicate upregulation in Group 2. Gray indicates that a median differential expression value was not calculated due to a low DEG count. (PDF) [file pone.0248202.s001.pdf]

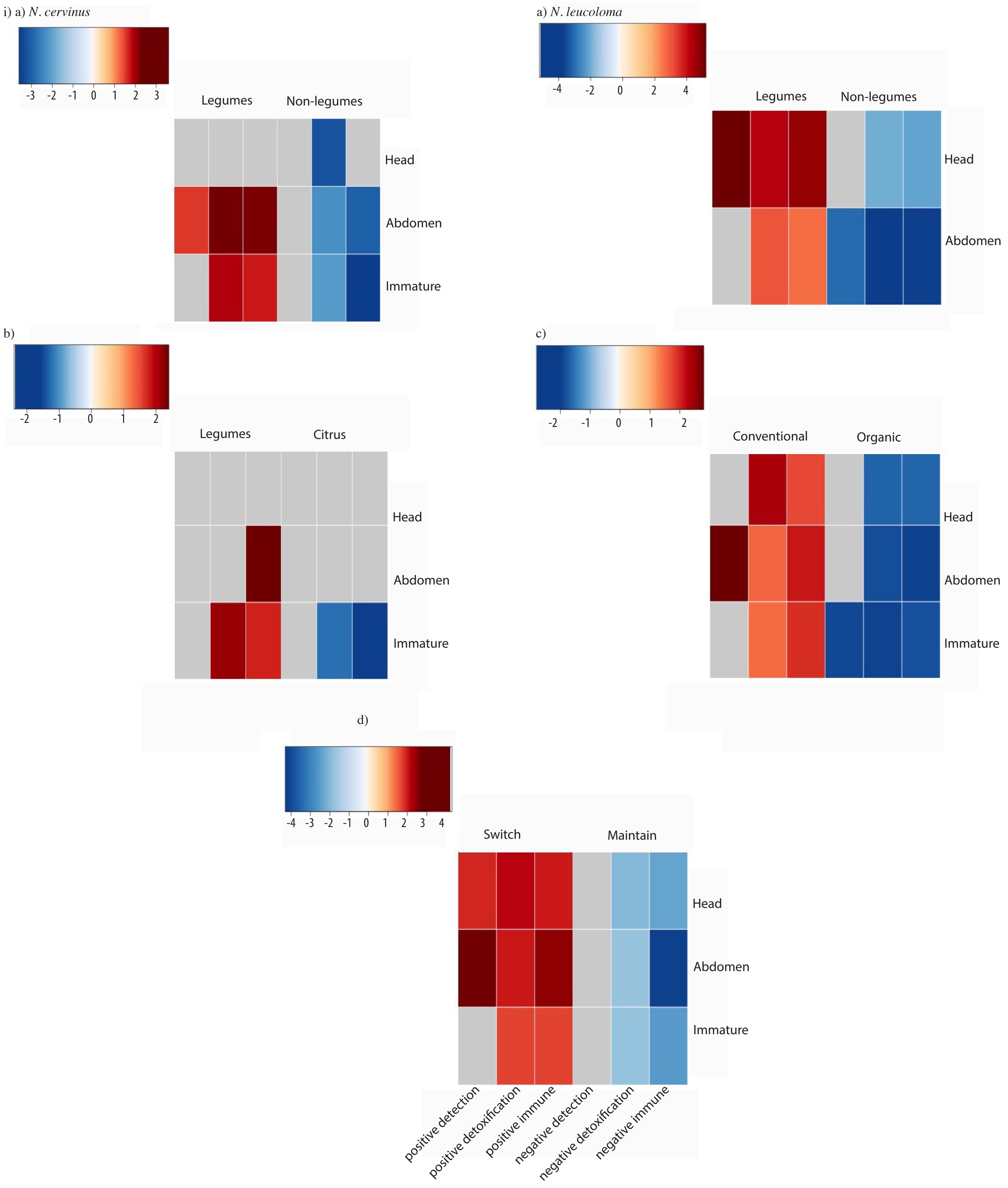
i) a) N. cervinus
-3 -2 -1 0 1 2 3
Legumes
Non-legumes
Head
Abdomen
Immature
a) N. leucoloma
-4 -2 0 2 4
Legumes
Non-legumes
Head
Abdomen
b)
-2 -1 0 1 2
Legumes
Citrus
Head
Abdomen
Immature
c)
-2 -1 0 1 2
Conventional
Organic
Head
Abdomen
Immature
d)
-4 -3 -2 -1 0 1 2 3 4
Switch
Maintain
Head
Abdomen
Immature
positive detection
positive detoxification
positive immune
negative detection
negative detoxification
negative immune

ii)

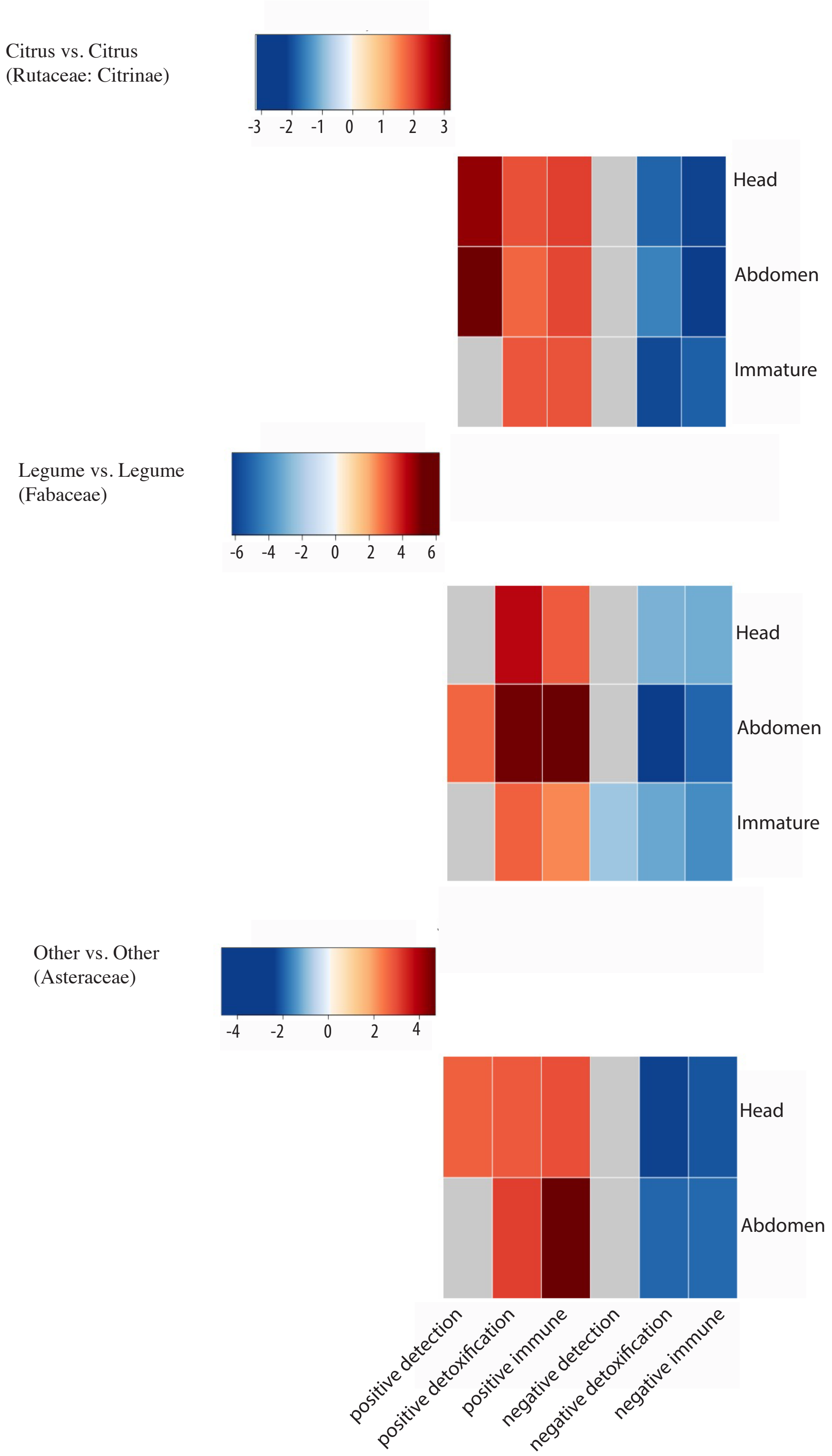
Citrus vs. Citrus
(Rutaceae: Citrinae)
-3 -2 -1 0 1 2 3
Head
Abdomen
Immature
Legume vs. Legume
(Fabaceae)
-6 -4 -2 0 2 4 6
Head
Abdomen
Immature
Other vs. Other
(Asteraceae)
-4 -2 0 2 4
Head
Abdomen
positive detection
positive detoxification
positive immune
negative detection
negative detoxification
negative immune
