# Supplementary material for: Host-specific gene expression as a tool for introduction success in Naupactus parthenogenetic weevils
Source: PLoS One. 2021 Jul 30;16(7):e0248202. doi: 10.1371/journal.pone.0248202 (PMC8323892; doi:10.1371/journal.pone.0248202)

i) Legume vs. Other

a) Head tissue

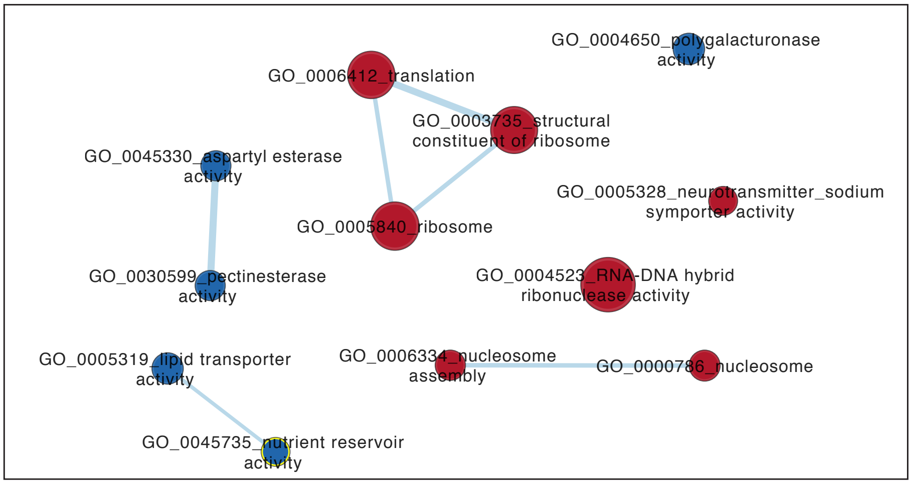


b) Abdomen tissue

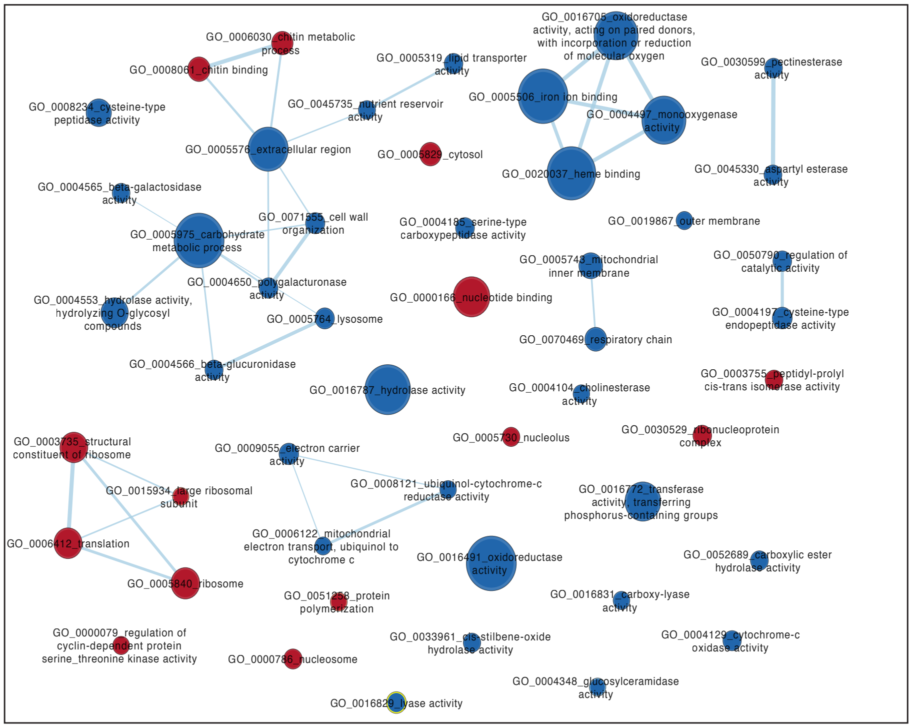


c) Immature tissue

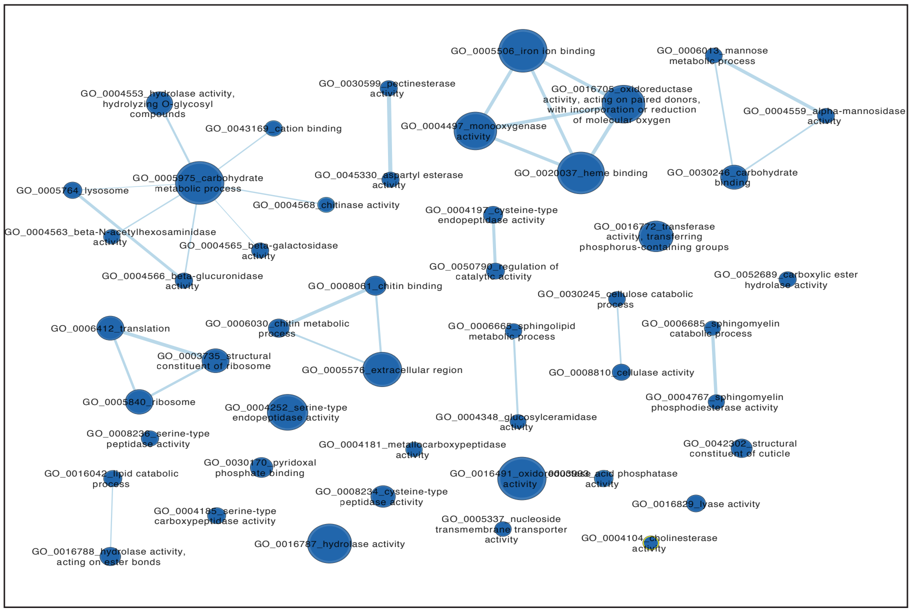

ii) Legume vs. Citrus

a) Head tissue

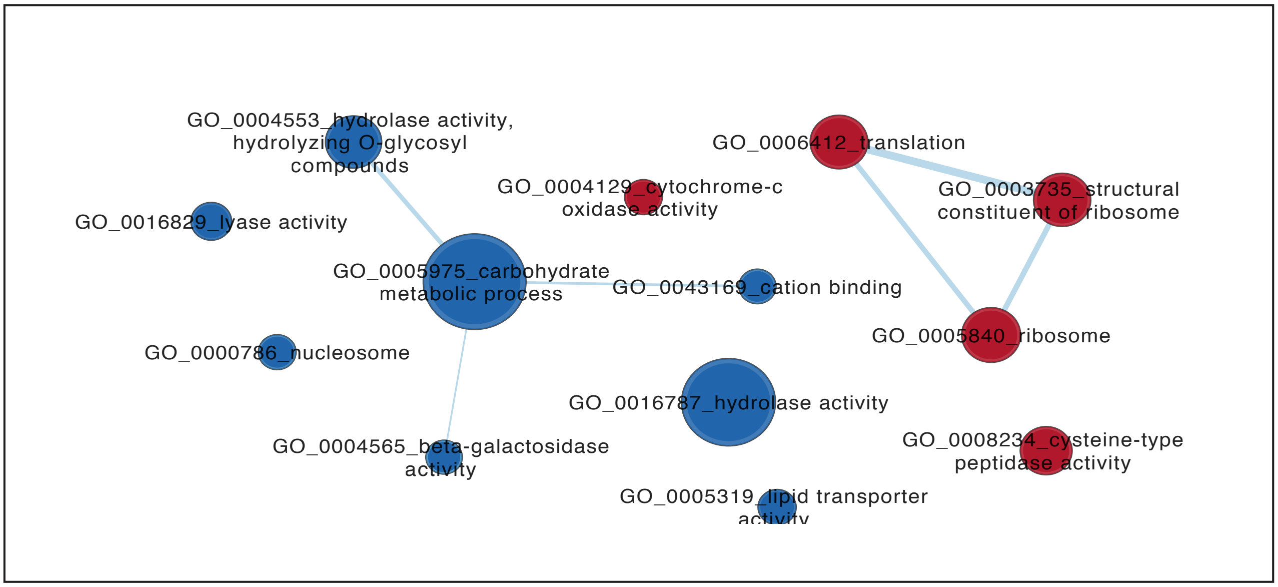


b) Abdomen tissue

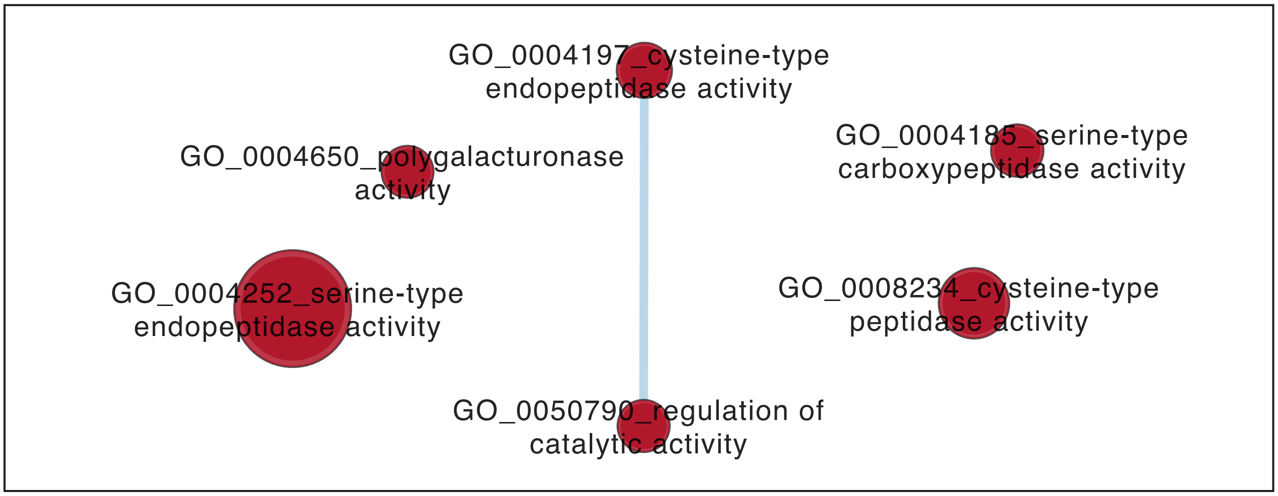


c) Immature tissue

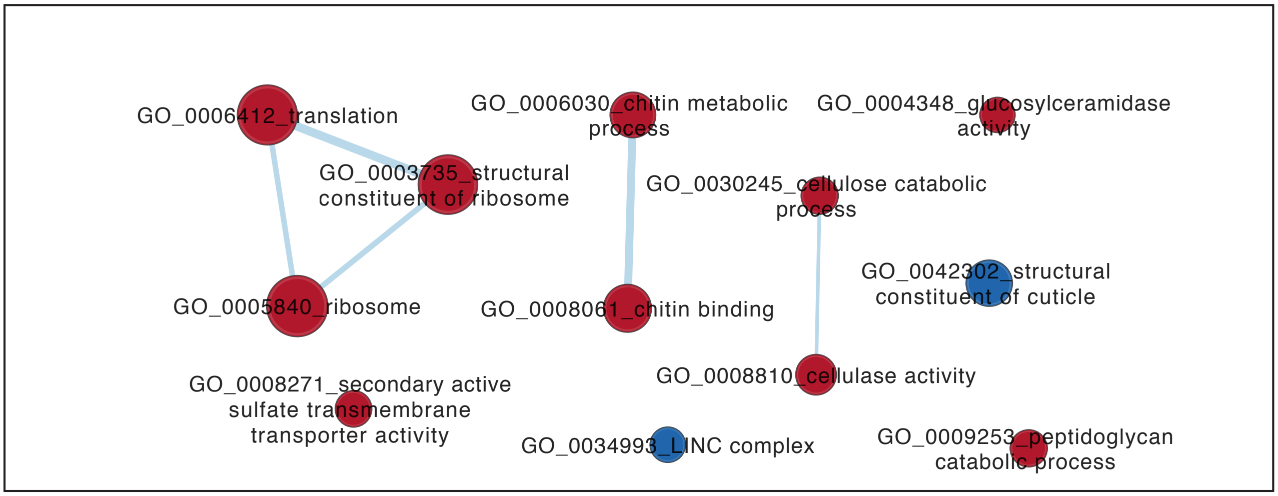

iii) Conventional vs. Organic

a) Head tissue

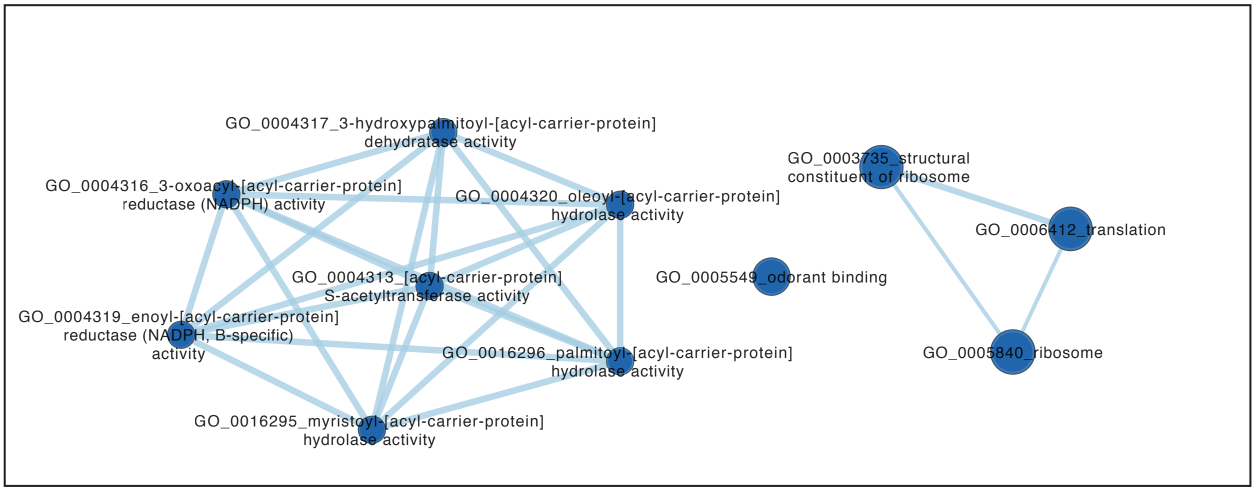


b) Abdomen tissue

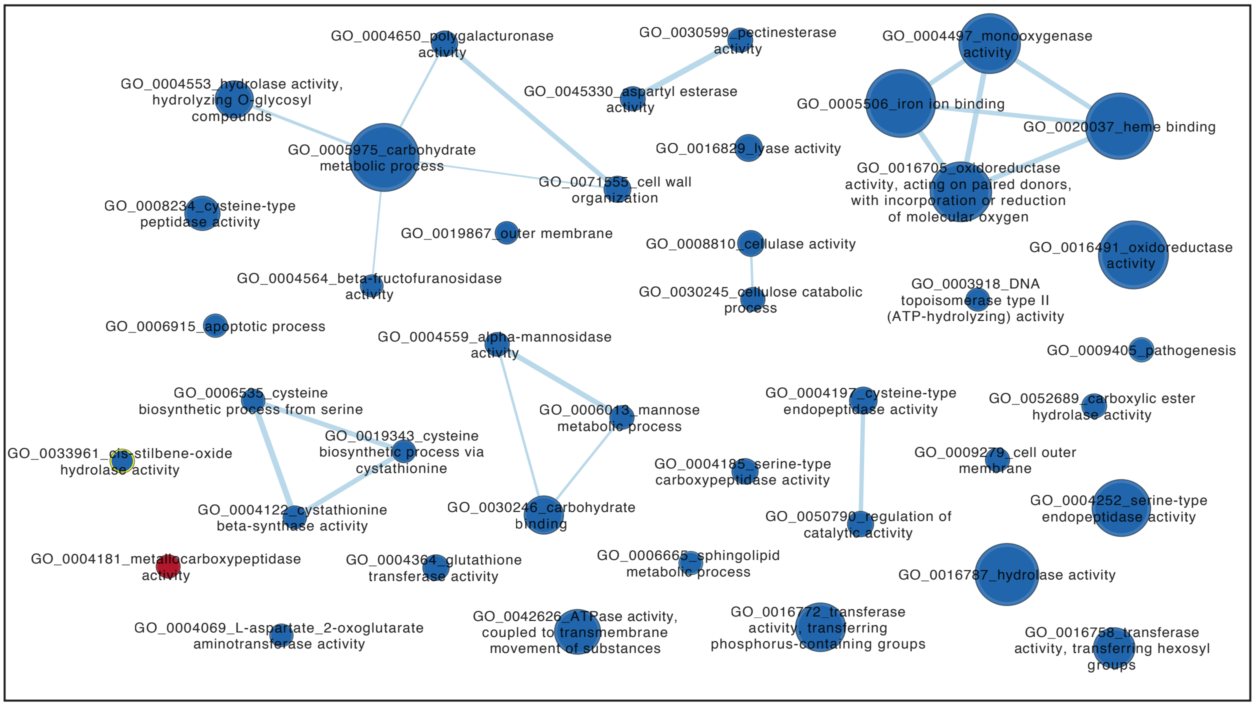


c) Immature tissue

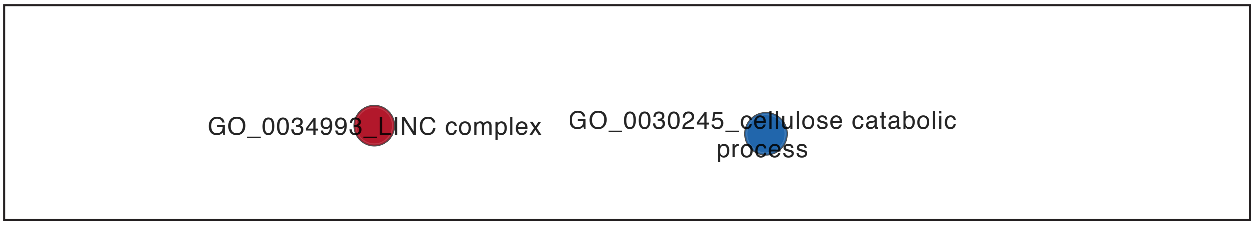

iv) Within family comparisons

a) Citrus vs. Citrus
Head tissue

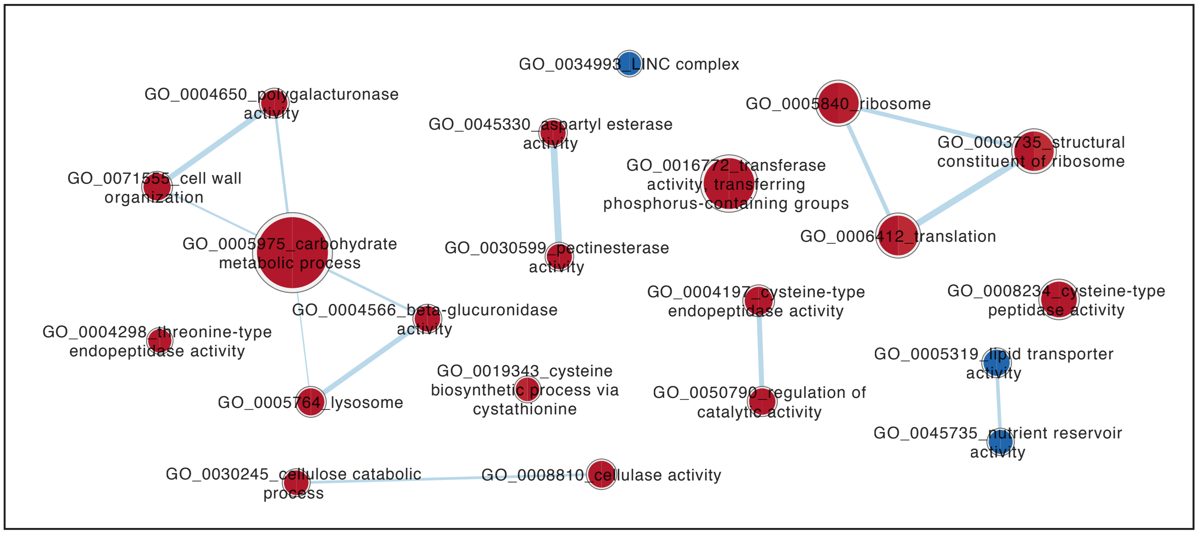


b) Citrus vs. Citrus
Abdomen tissue

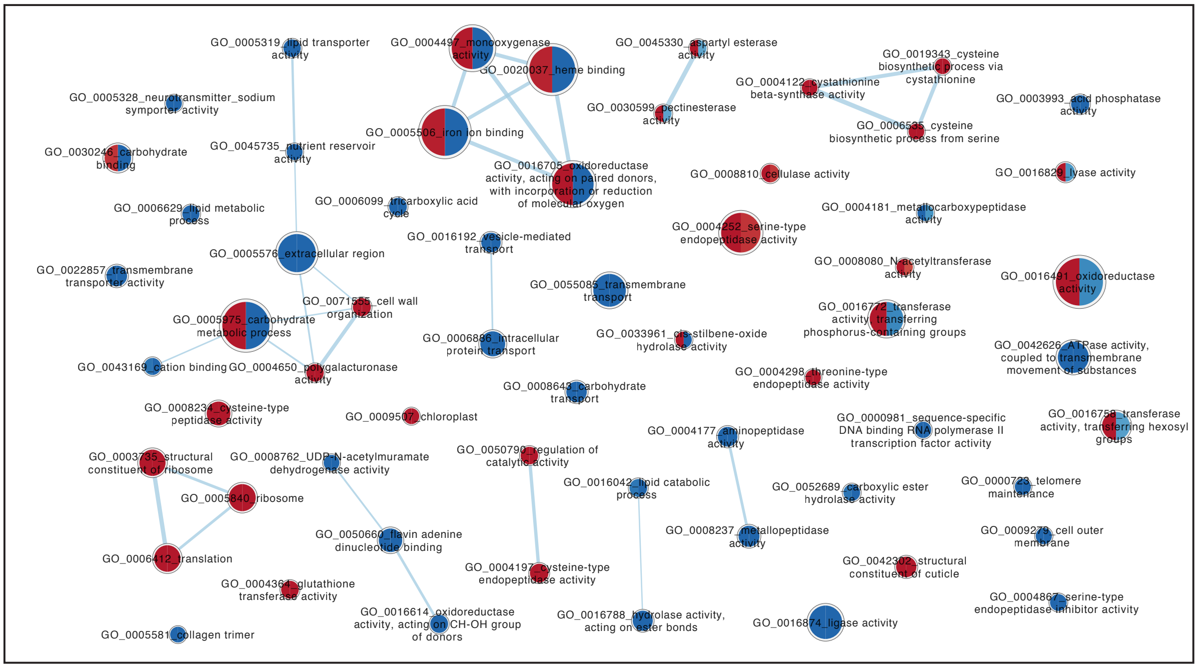


b) Citrus vs. Citrus
Immature tissue

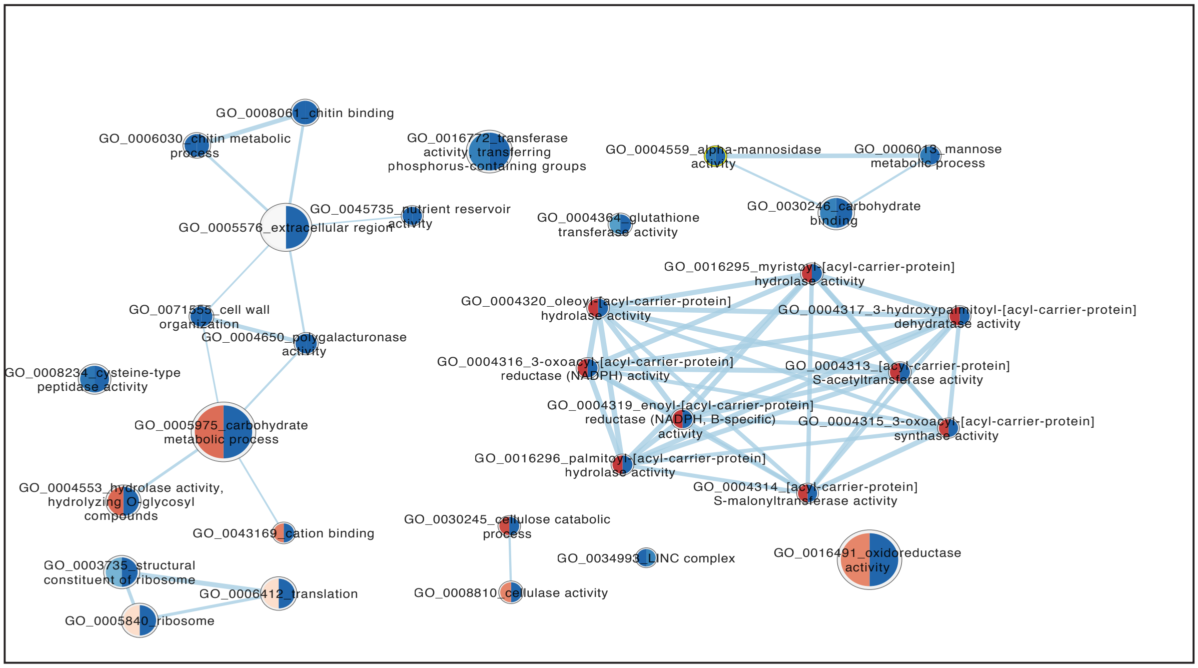

iv) Within family comparisons

d) Aster vs. Aster
Head tissue

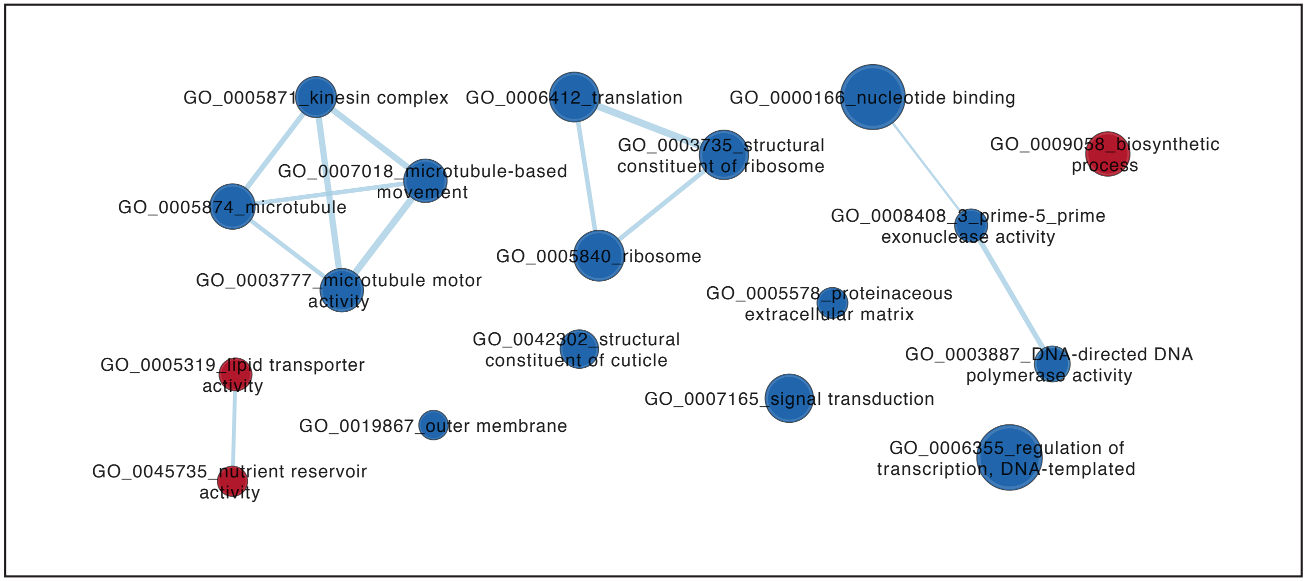


e) Aster vs. Aster
Abdomen tissue

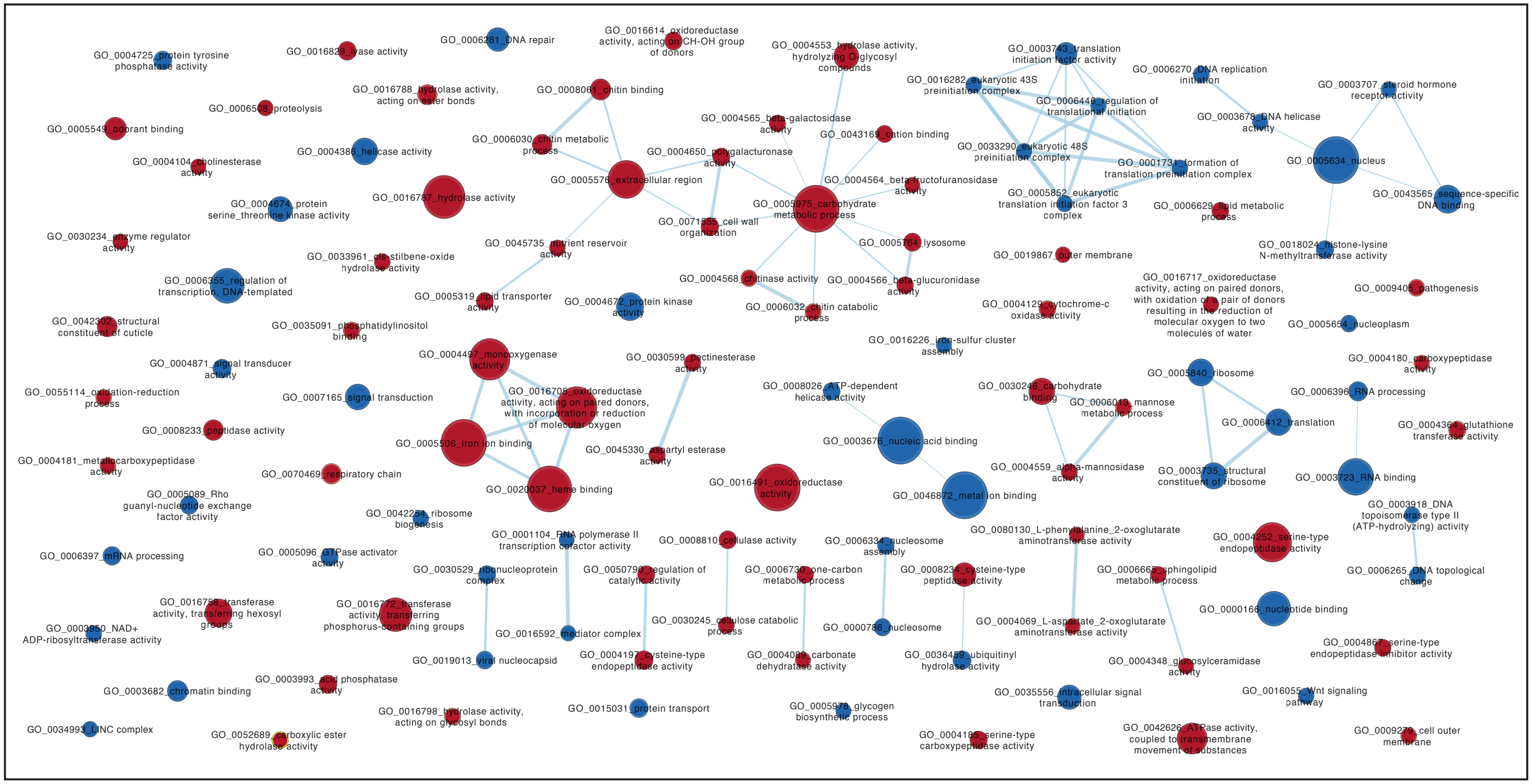

v) Switch vs. Maintain

a) Head tissue

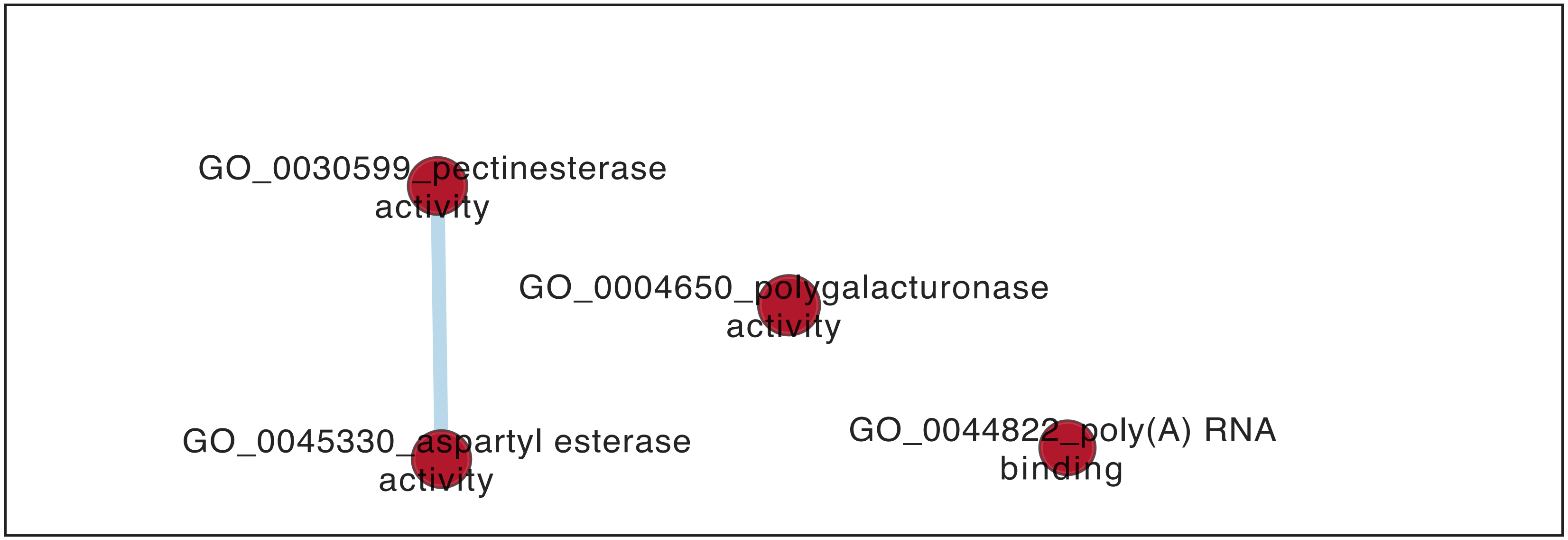


b) Adbomen tissue

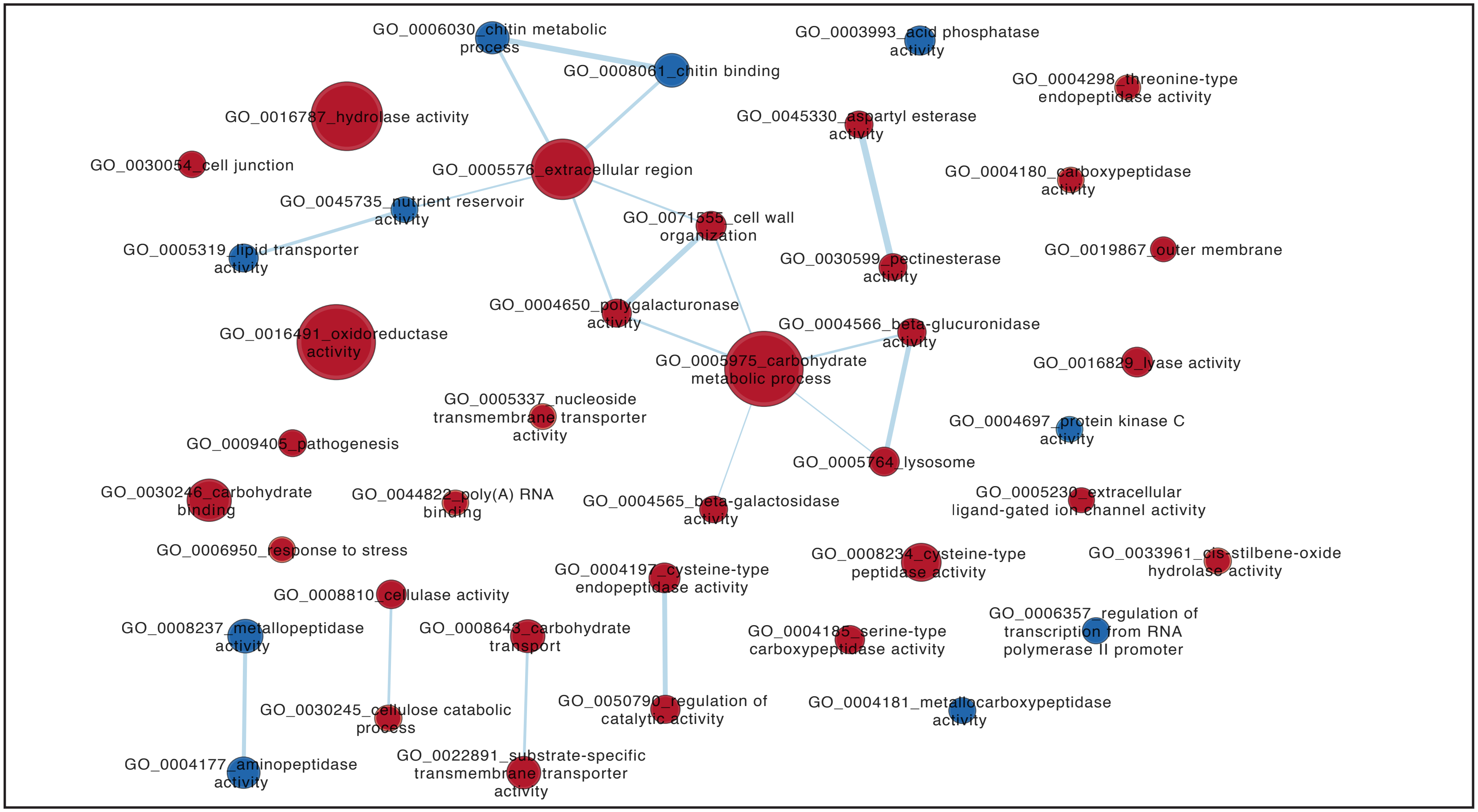

Supplement: S2 Fig — Gene Ontology (GO) term coloration in red indicates upregulation in G1, whereas blue coloration indicates upregulation in G2; the host plant listed first always corresponds to G1. (i) Weevil transcriptome comparisons while feeding on Legume vs. Other host plants: a) N. cervinus head tissue comparison (Comparison C25i/n). b) N. cervinus abdomen tissue comparison (Comparison C24i/n). c) N. cervinus immature tissue comparison (Comparison C56i). (ii) Weevil transcriptome comparisons while feeding on Legume vs. Citrus host plants: a) N. cervinus head tissue comparison (Comparison C66i). b) N. cervinus abdomen tissue comparison (Comparison C67i). (iii) Weevil transcriptome comparisons while feeding on Conventional vs. Organic host plants: a) N. cervinus head tissue comparison (Comparison C38i). b) N. cervinus abdomen tissue comparison (Comparison C39i). c) N. cervinus immature tissue comparison (Comparisons C40i). (iv) Weevil transcriptome comparisons while feeding on host plants within the same host-plant family: a) N. cervinus citrus-citrus head tissue comparison, (Comparisons C50i and C44i). b) N. cervinus citrus-citrus abdomen tissue comparison (Comparisons C51i and C45i). c) N. cervinus citrus-citrus immature tissue comparison (Comparisons C52i and C46i). Left hemisphere represents an organic orange vs. rough lemon comparison, whereas the right hemisphere represents a conventional orange vs. rough lemon comparison. d) N. cervinus aster-aster head tissue comparison (Comparison C27i/n). e) N. cervinus abdomen tissue comparison (Comparison C26i/n). (v) Switched vs. Maintained host plant weevil transcriptome comparisons: a) N. cervinus head tissue comparison (Comparison C69i). b) N. cervinus abdomen tissue comparison (Comparison C70i). (PDF) [file pone.0248202.s002.pdf]
