# Supplementary material for: Host-specific gene expression as a tool for introduction success in Naupactus parthenogenetic weevils
Source: PLoS One. 2021 Jul 30;16(7):e0248202. doi: 10.1371/journal.pone.0248202 (PMC8323892; doi:10.1371/journal.pone.0248202)
Supplement: S1 Table — General area indicates if the weevils were gathered from the introduced (INT) or native (NAT) range. Locality name and Coordinates provide locality details with location and state or province codes or names. Host plant indicates the plants where weevils were collected from and were maintained in those hosts while in the lab. When localities had multiple hosts, those are numbered and included in the lab sample code. Lab sample codes include locality code with species designation (C and L), host number in that locality (some localities yielded samples from multiple host plants), tissue (A: Head, B: Abdomen, I: Immature) and preparation number. For samples involved in the switch experiment, numbers in parentheses after the host label indicate switched to a new host plant (1) or maintained in the natal host plant (2) (for example: "Quin71C1(2)A1" denotes the first RNA preparation of head tissue from N. cervinus collected in FL on the one host present in that locality and maintained in that natal host). Comparisons indicates in which comparison groups those samples were included. Details of the comparisons are provided in S2 Table. (DOCX) [file pone.0248202.s003.docx]

**S1 Table. List of collection records and samples organized by area.** General area indicates if the weevils were gathered from the introduced (INT) or native (NA) range. Locality name and Coordinates provide locality details with location and state or province codes or names. Host plant indicates the plants where weevils were collected from and were maintained in those hosts while in the lab. When localities had multiple hosts, those are numbered and included in the lab sample code. Lab sample codes include locality code with species designation (C and L), host number in that locality (some localities yielded samples from multiple host plants), tissue (A: head, B: abdomen, I: immature) and preparation number. For samples involved in the switch experiment, numbers in parentheses after the host label indicate switched to a new host plant (1) or maintained in the natal host plant (2) (for example: "Quin71C1(2)A1" denotes the first RNA preparation of head tissue from *N. cervinus* collected in FL on the one host present in that locality and maintained in that natal host). Comparisons indicates in which comparison groups those samples were included. Details of the comparisons are provided in Supplementary Table 2.

| General Area | Locality name | Coordinates | Locality code | Host plant | Lab sample codes | Comparisons | Species |
| --- | --- | --- | --- | --- | --- | --- | --- |
| INT: USA: Southeast | Forsyth, GA | 33°02.272' N ; 083°55.55' W | For67C | Kudzu (Pueraria montana) | For67C1A1, For67C1B1, | Legume vs. Other; Legume vs. Citrus | *N. cervinus* |
|  | Byron, GA | 32°39.225' N ; 083°42.91' W | Byr68C | Asteraceae | Byr68C1A1, Byr68C1B1, Byr68C1I1 | Legume vs. Other; Asteraceae (HF) | *N. cervinus* |
|  | Post Street Park, Douglasville GA | 33°42.392' N ; 084°50.557' W | Post70C | Kudzu (Pueraria montana) | Post70C1A1, Post70C1B1, Post70C1I1 | Legume vs. Other; Legume vs. Citrus | *N. cervinus* |
|  | Post Street Park, Douglasville GA | 33°42.392' N ; 084°50.557' W | Post70L | Kudzu (Pueraria montana) | Post70L1A1, Post70L1B1 | Legume vs. Other; Fabaceae (HF) | *N. leucoloma* |
|  | Quincy, FL | 33°42.392' N ; 084°50.557' W | Quin71C | *Pawlonia tomentosa* (2) and Crepe myrtle (Lagerstroemia sp.) (4) | Quin71C2(1)A1, Quin71C2(2)A1, Quin71C2(1)B1, Quin71C2(2)B1, Quin71C2I1, Quin71C4I1 | Legume vs. Other; Switch vs. maintain | *N. cervinus* |
|  | Oleary, FL | 29°49.441' N ; 082°35.391' W | Olear72C | Kudzu (Pueraria montana) | Olear72C1A1, Olear72C1B1, Olear72C1I1 | Legume vs. Other; Asteraceae (HF) | *N. cervinus* |
|  | Fairhope, AL | 30°32.293' N ; 087°53.022' W | Fair74L | Soybean | Fair74L1A1, Fair74L1B1, Fair74L1I1 | Legume vs. Other; Fabaceae (HF) | *N. leucoloma* |
| INT: USA: West Coast | Kern Co., CA | 35°21.626' N ; 118°52.444' W | Ker_oneC | Oranges (Citrus sinensis “Valencia”), organic methods | Ker_oneC1A1, Ker_oneC1B1 | Legume vs. Citrus; Conventional vs. Organic, Rutaceae (HF) | *N. cervinus* |
|  | Kern Co., CA | 35°21.501' N ; 118°51.801' W | Ker_twoC | Oranges (Citrus sinensis “Valencia”), organic methods | Ker_twoC1I1 | Legume vs. Citrus; Conventional vs. Organic, Rutaceae (HF) | *N. cervinus* |
|  | Tulare Co., CA | 36°21.106' N ; 119°04.836' W | Tul_oneC | Oranges (Citrus sinensis “Valencia”), conventional methods | Tul_oneC1A1, Tul_oneC1B1, Tul_onetwoC1I1* | Legume vs. Citrus; Conventional vs. Organic, Rutaceae (HF) | *N. cervinus* |
|  | Tulare Co., CA | 36°19.987' N ; 119°05.603' W | Tul_twoC | Oranges (Citrus sinensis “Valencia”), conventional methods | Tul_twoC1A1, Tul_twoC1B1, Tul_onetwoC1I1* | Legume vs. Citrus; Conventional vs. Organic, Rutaceae (HF) | *N. cervinus* |
|  | Tulare Co., CA | 36°21.216' N ; 119°83.377' W | Tul_threeC (1,2,3,4,5,6) | varied hosts. 1: Pummelo; (Citrus maxima)2: Valencia oranges (Citrus sinensis “Valencia”); 3: Rubidoux mandarin; 4: Kao Pan Pummelo (Citrus maxima “Kao Pan”); 5: Tahitian and Sarawak Pummelo (Citrus maxima “Tahitian and Sarawak”); 6: UCLA and Vangasy rough lemon (Citrus jambhiri “UCLA and Vangasy” | Tul_threeC4A1, Tul_threeC4I2,  Tul_threeC6(1)A1, Tul_threeC6(2)A1, Tul_threeC6(1)B1, Tul_threeC6(2)B1, Tul_threeC6(1)I2, Tul_threeC6(2)I2, | Legume vs. Citrus; Rutaceae (HF); Switch vs. maintain | *N. cervinus* |
| NAT: Argentina: Rio de la Plata area | Pereyra Iraola, Buenos Aires | 34°50.96' S ; 058°08.13' W | Per76C | *Prunella vulgaris* | Per76C1A1; Per76C1B1 | Legume vs. Other | *N. cervinus* |
|  | Rosedal, Buenos Aires | 34°34.148' S ; 058°25.15' W | Ros77C | Caprifoleacea | Ros77C1A1; Ros77C1B1 | Legume vs. Other | *N. cervinus* |
|  | Rosedal, Buenos Aires | 34°34.148' S ; 058°25.15' W | Ros77L | *Poncirus trifoliata* | Ros77L1A1; Ros77L1B1 | Legume vs. Other | *N. leucoloma* |
|  | Ottamendi, Buenos Aires | 34°13.773' S ; 058°53.84' W | Otta78C | *Baccharis salisifolia* | Otta78C1A1; Otta78C1B1 | Legume vs. Other; Asteraceae (HF) | *N. cervinus* |
|  | Gualeguaychu, Entre Rios | 33°03.285' S ; 058°25.74' W | Ñan79C | *Baccharis sp.* | Ñan79C1A1; Ñan79C1B1 | Legume vs. Other; Asteraceae (HF) | *N. cervinus* |
|  | Gualeguaychu, Entre Rios | 32°58.354' S ; 058°27.78' W | Eli80L | *Soybean (Glycine max)* | Eli80L1A1 | Legume vs. Other; Fabaceae (HF) | *N. leucoloma* |
|  | Isla Talavera, Entre Rios | 34°04.830' S ; 058°59.79' W | Tala81C | *Baccharis sp.* | Tala81C1A1; Tala81C1B1 | Legume vs. Other; Asteraceae (HF) | *N. cervinus* |
|  | Solis, Buenos Aires | 34°18.760' S ; 059°17.209' W | Sol82L | Soybean *(Glycine max)* | Sol82L1A1 | Legume vs. Other | *N. leucoloma* |
