# Supplementary material for: Host-specific gene expression as a tool for introduction success in Naupactus parthenogenetic weevils
Source: PLoS One. 2021 Jul 30;16(7):e0248202. doi: 10.1371/journal.pone.0248202 (PMC8323892; doi:10.1371/journal.pone.0248202)
Supplement: S2 Table — Comparison names include species label: C = N. cervinus; L = N. leucoloma; number within that species and range: i = both sample groups originated from the introduced range; i/n = sample groups are from different ranges, one from introduced range and one from the native range; n = both sample groups originated from the native range. Comparison details include Species name, host plant, host plant groups, or experimental condition for each sample group. Comparison groups display lab sample codes as detailed in S1 Table, which also includes the geographic origin of each sample. (DOCX) [file pone.0248202.s004.docx]

**S2 Table. Details of each group of contrasts used for differential expression analysis organized by prediction.** Comparison names include species label: C = *N. cervinus*; L = *N. leucoloma;* number within that species and range: i = both sample groups originated from the introduced range; i/n = sample groups are from different ranges, one from introduced range and one from the native range; n = both sample groups originated from the native range. Comparison details include Species name, host plant, host plant groups, or experimental condition for each sample group. Comparison groups display lab sample codes as detailed in S1 Table, which also includes the geographic origin of each sample.

| **Prediction** | **Comparison Name** | **Comparison Details: Species/host plant or experimental condition (tissue)** | **Comp. Group 1** | **Comp. Group 2** |
| --- | --- | --- | --- | --- |
| Legume vs. Other | Comp. C17i/n | *N. cervinus/Kudzu/Prunella (head)* | For67C1A1  Post70C1A1 | Per76C1A1 |
|  | Comp. C21i/n | *N. cervinus/Kudzu/Caprifoleacea (head)* | For67C1A1  Post70C1A1 | Ros77C1A1 |
|  | Comp. C25i/n | *N. cervinus/Kudzu/Baccharis (head)* | For67C1A1  Post70C1A1 | Otta78C1A1  Ñan79C1A1  Tala81C1A1 |
|  | Comp. C3i | *N. cervinus/Kudzu/Asters (head)* | For67C1A1  Post70C1A1 | Byr68C1A1  Oleary72C1A1 |
|  | Comp. C5i | *N. cervinus/Asters/Kudzu (abdomen)* | Byr68C1B1  Oleary72C1B1 | For67C1B1  Post70C1B1 |
|  | Comp. C16i/n | *N. cervinus/Kudzu/Prunella (abdomen)* | For67C1B1  Post70C1B1 | Per76C1B1 |
|  | Comp. C20i/n | *N. cervinus/Kudzu/Caprifoleacea (abdomen)* | For67C1B1  Post70C1B1 | Ros77C1B1 |
|  | Comp. C24i/n | *N. cervinus/Kudzu/Baccharis (abdomen)* | For67C1B1  Post70C1B1 | Otta78C1B1  Ñan79C1B1  Tala81C1B1 |
|  | Comp. C56i | *N. cervinus/Kudzu/aster (immature)* | Post70C1I1 | Olear72C1I1  Byr68C1I1 |
|  | Comp. C57i | *N. cervinus/Kudzu/Paulownia (immature)* | Post70C1I1 | Quin71C2I1 |
|  | Comp. C58i | *N. cervinus/Kudzu/Crepe myrtle (immature)* | Post70C1I1 | Quin71C4I1 |
|  | Comp. L5i/n | *N. leucoloma/Soybean/Spiraea (head)* | Eli80L1A1  Fair74L1A1  Sol82L1A1 | Ros77L1A1 |
|  | Comp. L29i/n | *N. leucoloma/Spiraea/Kudzu (head)* | Ros77L1A1 | Post70L1A1 |
|  | Comp. L15n | *N. leucoloma/Spiraea/Soybean (head)* | Ros77L1A1 | Eli80L1A1  Sol82L1A1 |
|  | Comp. L28i/n | *N. leucoloma/Spiraea/Kudzu (abdomen)* | Ros77L1B1 | Post70L1B1 |
|  | Comp. L6i/n | *N. leucoloma/Soybean/Spiraea (abdomen)* | Post70L1B1  Fair74L1B1 | Ros77L1B1 |
| Legume vs. Citrus | Comp. C66i/n | *N. cervinus/Citrus/Legume (head)* | Ker_oneC1A1  Tul_twoC1A1  Tul_threeC4A1  Tul_threeC6(1)A1 | Post70C1A1  For67C1A1 |
|  | Comp. C67i/n | *N. cervinus/Citrus/Legume (abdomen)* | Ker_oneC1B1  Tul_twoC1B1  Tul_threeC4B1  Tul_threeC6(1)B1 | Post70C1B1  For67C1B1 |
|  | Comp. C68i/n | *N. cervinus/Citrus/Legume (immature)* | Ker_twoC2I1  Tul_onetwoC1I1  Tul_threeC4I2  Tul_threeC6(1)I2 | Post70C1I1 |
| Conventional vs. Organic | Comp. C38i | *N. cervinus/conventional oranges/organic oranges (head)* | Tul_twoC1A1 | Ker_oneC1A1 |
|  | Comp. C39i | *N. cervinus/conventional oranges/organic oranges (abdomen)* | Tul_twoC1B1 | Ker_oneC1B1 |
|  | Comp. C40i | *N. cervinus/conventional oranges/organic oranges (immature)* | Tul_onetwoC1I1 | Ker_twoC2I1 |
| HF: Asteraceae comparison | Comp. C27i/n | *N. cervinus/Baccharis/Aster (head)* | Otta78C1A1  Ñan79C1A1  Tala81C1A1 | Byr68C1A1  Oleary72C1A1 |
|  | Comp. C26i/n | *N. cervinus/Baccharis/Aster (abdomen)* | Otta78C1B1  Ñan79C1B1  Tala81C1B1 | Byr68C1B1  Oleary72C1B1 |
| HF: Fabaceae comparison | Comp. L30i/n | *N. leucoloma/Kudzu/Soybean (head)* | Post70L1A1 | Eli80L1A1  Sol82L1A1 |
|  | Comp. L1i | *N. leucoloma/Kudzu/Soybean (head)* | Post70L1A1 | Fair74L1A1 |
|  | Comp. L2i | *N. leucoloma/Kudzu/Soybean (abdomen)* | Post70L1B1 | Fair74L1B1 |
|  | Comp. L62i | *N. leucoloma/kudzu/soybean (immature)* | Post70L1I1 | Fair74L1I1 |
| HF: Rutaceae comparison | Comp. C47i | *N. cervinus/organic oranges/Pummelos (CRC, Kao Pan, Tahitian/Sarawak) (head)* | Ker_oneC1A1 | Tul_threeC4A1 |
|  | Comp. C50i | *N. cervinus/organic oranges/UCLA and Vangassay rough lemon (head)* | Ker_oneC1A1 | Tul_threeC6(1)A1 |
|  | Comp. C53i | *N. cervinus/Pummelos (CRC, Kao Pan, Tahitian/Sarawak)/UCLA and Vangassay rough lemon (head)* | Tul_threeC4A1 | Tul_threeC6(1)A1 |
|  | Comp. C41i | *N. cervinus/conventional oranges/Pummelos (CRC, Kao Pan, Tahitian/Sarawak) (head)* | Tul_twoC1A1 | Tul_threeC4A1 |
|  | Comp. C44i | *N. cervinus/conventional oranges/UCLA and Vangassay rough lemon (head)* | Tul_twoC1A1 | Tul_threeC6(1)A1 |
|  | Comp. C48i | *N. cervinus/organic oranges/Pummelos (CRC, Kao Pan, Tahitian/Sarawak) (abdomen)* | Ker_oneC1B1 | Tul_threeC4B1 |
|  | Comp. C51i | *N. cervinus/organic oranges/UCLA and Vangassay rough lemon (abdomen)* | Ker_oneC1B1 | Tul_threeC6(1)B1 |
|  | Comp. C54i | *N. cervinus/Pummelos (CRC, Kao Pan, Tahitian/Sarawak)/UCLA and Vangassay rough lemon (abdomen)* | Tul_threeC4B1 | Tul_threeC6(1)B1 |
|  | Comp. C42i | *N. cervinus/conventional oranges/Pummelos (CRC, Kao Pan, Tahitian/Sarawak) (abdomen)* | Tul_twoC1B1 | Tul_threeC4B1 |
|  | Comp. C45i | *N. cervinus/conventional oranges/UCLA and Vangassay rough lemon (abdomen)* | Tul_twoC1B1 | Tul_threeC6(1)B1 |
|  | Comp. C49i | *N. cervinus/organic oranges/Pummelos (CRC, Kao Pan, Tahitian/Sarawak) (immature)* | Ker_twoC2I1 | Tul_threeC4I2 |
|  | Comp. C52i | *N. cervinus/organic oranges/UCLA and Vangassay rough lemon (immature)* | Ker_twoC2I1 | Tul_threeC6(1)I2 |
|  | Comp. C43i | *N. cervinus/conventional oranges/Pummelos (CRC, Kao Pan, Tahitian/Sarawak) (immature)* | Tul_onetwoC1I1 | Tul_threeC4I2 |
|  | Comp. C46i | *N. cervinus/conventional oranges/UCLA and Vangassay rough lemon (immature)* | Tul_onetwoC1I1 | Tul_threeC6(1)I2 |
|  | Comp. C55i | *N. cervinus/Pummelos (CRC, Kao Pan, Tahitian/Sarawak)/UCLA and Vangassay rough lemon (immature)* | Tul_threeC4I2 | Tul_threeC6(1)I2 |
| Switch vs. Maintain | Comp. C2i | *N. cervinus/Sweet potato/Paulownia (head)* | Quin71C1(1)A1 | Quin71C1(2)A1 |
|  | Comp.C4i | *N. cervinus/Sweet potato/Paulownia (abdomen)* | Quin71C1(1)B1 | Quin71C1(2)B1 |
|  | Comp. C69i | *N. cervinus/Sweet potato/Rough lemon (head)* | Tul_threeC6(1)A1 | Tul_threeC6(2)A1 |
|  | Comp. C70i | *N. cervinus/Sweet potato/Rough lemon (abdomen)* | Tul_threeC6(1)B1 | Tul_threeC6(2)B1 |
|  | Comp. C71i | *N. cervinus/Sweet potato/Rough lemon (immature)* | Tul_threeC6(1)I2 | Tul_threeC6(2)I2 |
