# Supplementary material for: Host-specific gene expression as a tool for introduction success in Naupactus parthenogenetic weevils
Source: PLoS One. 2021 Jul 30;16(7):e0248202. doi: 10.1371/journal.pone.0248202 (PMC8323892; doi:10.1371/journal.pone.0248202)
Supplement: S3 Table — Reads after quality filtering denote the total quantity of reads after read trim filtering in Trinity; reads mapped indicate the total number of reads successfully mapped to the de novo transcriptome assembly of that species. (DOCX) [file pone.0248202.s005.docx]

**S3 Table. Quality-filtered read mapping for each sample included in downstream comparisons.** Reads after quality filtering denote the total quantity of reads after Trimmomatic read filtering in Trinity; reads mapped indicate the total number of reads successfully mapped to the *de novo*  transcriptome assembly of that species.

| **Sample** | **Species assembly** | **Reads after quality filtering** | **Reads mapped** | **% reads mapped** |
| --- | --- | --- | --- | --- |
| Byr68C1A1 | *N. cervinus* | 16,645,779 | 9,238,910 | 54.71% |
| Byr68C1B1 | *N. cervinus* | 11,501,546 | 4,625,134 | 40.21% |
| Byr68C1I1 | *N. cervinus* | 30,500,412 | 18,855,355 | 61.82% |
| Eli80L1A1 | *N. leucoloma* | 28,458,229 | 15,378,827 | 54.04% |
| Fair74L1A1 | *N. leucoloma* | 74,729,065 | 46,026,536 | 61.59% |
| Fair74L1B1 | *N. leucoloma* | 48,859,683 | 37,406,678 | 76.56% |
| Fair74L1I1 | *N. leucoloma* | 29,985,492 | 11,613,381 | 38.73% |
| For67C1A1 | *N. cervinus* | 80,756,243 | 58,766,318 | 72.77% |
| For67C1B1 | *N. cervinus* | 79,409,647 | 55,429,134 | 69.80% |
| Ker_oneC1A1 | *N. cervinus* | 34,532,904 | 24,487,282 | 70.91% |
| Ker_oneC1B1 | *N. cervinus* | 47,608,534 | 31,216,916 | 65.57% |
| Ker_twoC2I1 | *N. cervinus* | 30,952,875 | 19,732,458 | 63.75% |
| Ñan79C1A1 | *N. cervinus* | 26,341,807 | 16,703,340 | 63.41% |
| Ñan79C1B1 | *N. cervinus* | 38,166,913 | 16,320,172 | 42.76% |
| Olear72C1I1 | *N. cervinus* | 27,632,662 | 5,772,463 | 20.89% |
| Oleary72C1A1 | *N. cervinus* | 6,666,371 | 240,901 | 3.61% |
| Oleary72C1B1 | *N. cervinus* | 14,453,430 | 9,255,163 | 64.03% |
| Otta78C1A1 | *N. cervinus* | 28,740,859 | 18,399,898 | 64.02% |
| Otta78C1B1 | *N. cervinus* | 43,537,805 | 23,323,202 | 53.57% |
| Per76C1A1 | *N. cervinus* | 24,725,556 | 17,003,765 | 68.77% |
| Per76C1B1 | *N. cervinus* | 37,861,824 | 13,475,023 | 35.59% |
| Post70C1A1 | *N. cervinus* | 18,073,097 | 13,006,476 | 71.97% |
| Post70C1B1 | *N. cervinus* | 13,496,902 | 7,817,463 | 57.92% |
| Post70C1I1 | *N. cervinus* | 26,713,255 | 17,334,231 | 64.89% |
| Post70L1A1 | *N. leucoloma* | 21,995,741 | 14,237,533 | 64.73% |
| Post70L1B1 | *N. leucoloma* | 17,906,568 | 10,277,422 | 57.39% |
| Post70L1I1 | *N. leucoloma* | 21,349,808 | 11,259,889 | 52.74% |
| Quin71C1(1)A1 | *N. cervinus* | 16,745,952 | 10,880,968 | 64.98% |
| Quin71C1(1)B1 | *N. cervinus* | 21,175,854 | 13,939,995 | 65.83% |
| Quin71C1(2)A1 | *N. cervinus* | 18,179,581 | 12,334,230 | 67.85% |
| Quin71C1(2)B1 | *N. cervinus* | 18,434,502 | 12,159,820 | 65.96% |
| Quin71C2I1 | *N. cervinus* | 22,717,600 | 14,025,846 | 61.74% |
| Quin71C4I1 | *N. cervinus* | 29,006,445 | 19,338,597 | 66.67% |
| Ros77C1A1 | *N. cervinus* | 23,351,469 | 12,605,123 | 53.98% |
| Ros77C1B1 | *N. cervinus* | 27,241,140 | 14,026,463 | 51.49% |
| Ros77L1A1 | *N. leucoloma* | 37,891,426 | 21,757,257 | 57.42% |
| Ros77L1B1 | *N. leucoloma* | 28,723,883 | 13,233,093 | 46.07% |
| Sol82L1A1 | *N. leucoloma* | 22,952,393 | 11,262,739 | 49.07% |
| Tala81C1A1 | *N. cervinus* | 35,247,069 | 22,959,941 | 65.14% |
| Tala81C1B1 | *N. cervinus* | 23,045,100 | 13,921,545 | 60.41% |
| Tul_onetwoC1I1 | *N. cervinus* | 18,983,618 | 13,106,290 | 69.04% |
| Tul_threeC4A1 | *N. cervinus* | 32,385,943 | 22,135,792 | 68.35% |
| Tul_threeC4B1 | *N. cervinus* | 36,769,045 | 23,822,664 | 64.79% |
| Tul_threeC4I2 | *N. cervinus* | 35,081,762 | 22,697,900 | 64.70% |
| Tul_threeC6(1)A1 | *N. cervinus* | 36,963,879 | 26,854,258 | 72.65% |
| Tul_threeC6(1)B1 | *N. cervinus* | 32,567,100 | 21,917,658 | 67.30% |
| Tul_threeC6(1)I2 | *N. cervinus* | 28,882,238 | 19,310,664 | 66.86% |
| Tul_threeC6(2)A1 | *N. cervinus* | 34,445,364 | 22,423,932 | 65.10% |
| Tul_threeC6(2)B1 | *N. cervinus* | 35,776,805 | 22,911,466 | 64.04% |
| Tul_threeC6(2)I2 | *N. cervinus* | 30,327,239 | 19,785,491 | 65.24% |
| Tul_twoC1A1 | *N. cervinus* | 29,101,219 | 19,832,481 | 68.15% |
| Tul_twoC1B1 | *N. cervinus* | 34,868,501 | 23,501,370 | 67.40% |
