# Supplementary material for: Host-specific gene expression as a tool for introduction success in Naupactus parthenogenetic weevils
Source: PLoS One. 2021 Jul 30;16(7):e0248202. doi: 10.1371/journal.pone.0248202 (PMC8323892; doi:10.1371/journal.pone.0248202)
Supplement: S4 Table — Significantly enriched GO terms are displayed by hypothesis, species and tissue in decreasing order of normalized enrichment score (NES). Within each contrast, the numbers of significantly enriched GO terms and the direction of enrichment are indicated (enriched G1/enriched G2) together with the number of connections between GO terms in each direction produced by Cytoscape (positive/negative) and the location of the enrichment map within S2 Fig. MF, CC, BP indicate which category each enriched GO term belongs to (molecular function, cellular component, biological process) and the number after that indicates how many genes are in that set. Numbers in brackets after each GO term indicate values of (NES) and false discovery rate q-value (FDR). (DOCX) [file pone.0248202.s006.docx]

**S4 Table. Summary of significantly enriched GO terms derived from Enrichment Maps for each comparison and tissue.** Significantly enriched GO terms are displayed by hypothesis, species and tissue in decreasing order of Normalized enrichment score (NES). Within each contrast, the numbers of significantly enriched GO terms and the direction of enrichment are indicated (enriched G1/enriched G2) together with the number of connections between GO terms in each direction produced by Cytoscape (positive/negative) and the location of the enrichment map within S2 Fig. MF, CC, BP indicate which category each enriched GO term belongs to (molecular function, cellular component, biological process) and the number after that indicates how many genes are in that set. Numbers in brackets after each GO term indicate values of (NES) and false discovery rate q-value (FDR).

| Hypothesis |  | Head | Abdomen | Immature |
| --- | --- | --- | --- | --- |
| Legume vs. Other | # of enriched GO terms | 12 (7/5) | 27(10,17) | 18(0/18) |
|  | Enriched # of connections | 5/4 (S2 ia) | 6/20 (S2 ib) | 0/33 (S2 ic) |
|  | Positively enriched in G1, relative to G2: GO terms | GO_0000786_NUCLEOSOME: CC, 24 [2.77; 0]  GO_0003735_STRUCTURAL CONSTITUENT OF RIBOSOME: MF, 127 [2.58; 0]  GO_0006412_TRANSLATION: BP, 130 [2.51; 0]  GO_0006334_NUCLEOSOME ASSEMBLY: BP, 18 [2.34; 0.001]  GO_0005840_RIBOSOME: CC, 140 [2.25; 0.002]  GO_0004523_RNA-DNA HYBRID RIBONUCLEASE ACTIVITY: MF, 95 [2.10; 0.01]  GO_0005328_NEUROTRANSMITTER_SODIUM SYMPORTER ACTIVITY: MF, 13 [2.10; 0.01] | GO_0006412_TRANSLATION: BP, 130 [2.10; 0]  GO_0003735_STRUCTURAL CONSTITUENT OF RIBOSOME: MF, 127 [2.10; 0]  GO_0005840_RIBOSOME: CC, 140 [2.04; 2.71E-04]  GO_0005730_NUCLEOLUS: CC, 21 [1.93; 0.003]  GO_0006030_CHITIN METABOLIC PROCESS: BP, 68 [1.77; 0.04]  GO_0000786_NUCLEOSOME: CC, 24 [1.76; 0.03]  GO_0000166_NUCLEOTIDE BINDING: MF, 182 [1.75; 0.03]  GO_0000079_REGULATION OF CYCLIN-DEPENDENT PROTEIN SERINE_THREONINE KINASE ACTIVITY: BP, 14 [1.75; 0.03]  GO_0008061_CHITIN BINDING: MF, 76 [1.75; 0.03]  GO_0015934_LARGE RIBOSOMAL SUBUNIT: CC, 10 [1.70; 0.04] | N/A |
|  | Negatively enriched in G1, relative to G2: GO terms | GO_0030599_PECTINESTERASE ACTIVITY: MF, 29 [-2.07; 0]  GO_0045330_ASPARTYL ESTERASE ACTIVITY: MF, 29 [-2.06; 0]  GO_0004650_POLYGALACTURONASE ACTIVITY: MF, 33 [-2.04; 0]  GO_0045735_NUTRIENT RESERVOIR ACTIVITY: MF, 18 [-1.93; 6.28 E-04]  GO_0005319_LIPID TRANSPORTER ACTIVITY: MF, 35 [-1.73; 0.04] | GO_0004650_POLYGALACTURONASE ACTIVITY: MF, 33 [-3.20; 0]  GO_0045735_NUTRIENT RESERVOIR ACTIVITY: MF, 18 [-2.87; 0]  GO_0045330_ASPARTYL ESTERASE ACTIVITY: MF, 29 [-2.79; 0]  GO_0030599_PECTINESTERASE ACTIVITY: MF, 29 [-2.79; 0]  GO_0005319_LIPID TRANSPORTER ACTIVITY: MF, 35 [-2.60; 0]  GO_0050790_REGULATION OF CATALYTIC ACTIVITY: BP, 39 [-2.54; 0]  GO_0004197_CYSTEINE-TYPE ENDOPEPTIDASE ACTIVITY: MF, 50 [-2.52; 0]  GO_0004566_BETA-GLUCURONIDASE ACTIVITY: BP, 23 [-2.49; 0]  GO_0005975_CARBOHYDRATE METABOLIC PROCESS: BP, 369 [-2.47; 0]  GO_0004497_MONOOXYGENASE ACTIVITY: MF, 269 [-2.40; 1.15E-04]  GO_0020037_HEME BINDING: MF, 309 [-2.31; 5.43E-04]  GO_0016491_OXIDOREDUCTASE ACTIVITY: MF, [-2.31; 5.07E-04]  GO_0005506_IRON ION BINDING: MF, [-2.29; 6.79E-04]  GO_0016787_HYDROLASE ACTIVITY: MF, [-2.40; 1.15E-04]  GO_0016705_OXIDOREDUCTASE ACTIVITY, ACTING ON PAIRED DONORS, WITH INCORPORATION OR REDUCTION OF MOLECULAR OXYGEN: MF, 269 [-2.31; 1.15E-04]  GO_0005576_EXTRACELLULAR REGION: CC, 256 [-2.01; 0.009]  GO_0016772_TRANSFERASE ACTIVITY, TRANSFERRING PHOSPHORUS-CONTAINING GROUPS MF, 192 [-2.17; 0.002] | GO_0006030_CHITIN METABOLIC PROCESS: BP, 57 [-2.28; 0]  GO_0005975_CARBOHYDRATE METABOLIC PROCESS: BP, 424 [-2.24; 0]  GO_0008061_CHITIN BINDING: MF, 87 [-2.24; 0]  GO_0004197_CYSTEINE-TYPE ENDOPEPTIDASE ACTIVITY: MF, 53 [-2.21; 0]  GO_0005576_EXTRACELLULAR REGION: CC, 287 [-2.21; 0]  GO_0008234_CYSTEINE-TYPE PEPTIDASE ACTIVITY: MF, 116 [-2.15; 0]  GO_0004553_HYDROLASE ACTIVITY, HYDROLYZING O-GLYCOSYL COMPOUNDS: MF, 129 [ -2.14; 0]  GO_0004252_SERINE-TYPE ENDOPEPTIDASE ACTIVITY: MF, 307 [ -2.14; 0]  GO_0050790_REGULATION OF CATALYTIC ACTIVITY: BP, 42 [-2.13; 0]  GO_0008810_CELLULASE ACTIVITY: MF, 43 [-2.04; 0]  GO_0016491_OXIDOREDUCTASE ACTIVITY: MF, 436 [-1.92; 8.57E-05]  GO_0020037_HEME BINDING: MF, 362 [-1.67; 0.009]  GO_0005506_IRON ION BINDING: MF, 374 [-1.65; 0.01]  GO_0016787_HYDROLASE ACTIVITY: MF, 323 [-2.00; 0]  GO_0016705_OXIDOREDUCTASE ACTIVITY, ACTING ON PAIRED DONORS, WITH INCORPORATION OR REDUCTION OF MOLECULAR OXYGEN: MF, 313 [-1.65; 0.01]  GO_0004497_MONOOXYGENASE ACTIVITY: MF, 314 [-1.64; 0.01]  GO_0016772_TRANSFERASE ACTIVITY, TRANSFERRING PHOSPHORUS-CONTAINING GROUPS: MF, 219 [-2.19; 0.002] |
| Legume vs. Citrus | # of enriched GO terms | 13 (5/8) | 6 (6/0) | 12 (10/2) |
|  | Enriched # of connections | 3/4 (S2 iia) | 2/0 (S2 iib) | 7/0 (S2 iic) |
|  | Positively enriched in G1, relative to G2: GO terms | GO_0003735_STRUCTURAL CONSTITUENT OF RIBOSOME: MF, 118 [0.52; 2.13; 0.001]  GO_0006412_TRANSLATION: BP, 121 [2.10; 0.002]  GO_0005840_RIBOSOME: CC, 127 [1.99; 0.01]  GO_0008234_CYSTEINE-TYPE PEPTIDASE ACTIVITY: MF, 58 [1.93; 0.02]  GO_0004129_CYTOCHROME-C OXIDASE ACTIVITY: MF, 25 [1.91; 0.02] | GO_0004650_POLYGALACTURONASE ACTIVITY: MF, 29 [2.06; 0]  GO_0008234_CYSTEINE-TYPE PEPTIDASE ACTIVITY: MF, 32 [2.05; 0]  GO_0050790_REGULATION OF CATALYTIC ACTIVITY: BP, 77 [2.03; 0]  GO_0004185_SERINE-TYPE CARBOXYPEPTIDASE ACTIVITY: MF, 37 [1.79; 0.01]  GO_0004197_CYSTEINE-TYPE ENDOPEPTIDASE ACTIVITY: MF, 23 [1.79; 0.01]  GO_0004252_SERINE-TYPE ENDOPEPTIDASE ACTIVITY: MF, 43 [1.76; 0.02] | GO_0006412_TRANSLATION: BP, 144 [1.84; 0.001]  GO_0003735_STRUCTURAL CONSTITUENT OF RIBOSOME: MF, 141 [1.83; 0.001]  GO_0005840_RIBOSOME: CC, 153 [1.82; 9.15E-04]  GO_0008061_CHITIN BINDING: MF, 87 [1.81; 9.19E-04]  GO_0006030_CHITIN METABOLIC PROCESS: BP, 77 [1.76; 0.004]  GO_0008810_CELLULASE ACTIVITY: MF, 43 [0.69; 1.72; 0.01]  GO_0009253_PEPTIDOGLYCAN CATABOLIC PROCESS: BP, 23 [0.76; 1.70; 0.01]  GO_0030245_CELLULOSE CATABOLIC PROCESS: BP, 20 [1.68; 0.02]  GO_0008271_SECONDARY ACTIVE SULFATE TRANSMEMBRANE TRANSPORTER ACTIVITY: MF, 17 [1.65; 0.03]  GO_0004348_GLUCOSYLCERAMIDASE ACTIVITY: MF, 12 [1.63; 0.04] |
|  | Negatively enriched in G1, relative to G2: GO terms | GO_0005975_CARBOHYDRATE METABOLIC PROCESS: BP, 214 [-1.99; 0.006]  GO_0004553_HYDROLASE ACTIVITY, HYDROLYZING O-GLYCOSYL COMPOUNDS: MF, 64 [-1.97; 0.005]  GO_0016829_LYASE ACTIVITY: MF, 28 [-1.96; 0.004]  GO_0043169_CATION BINDING: MF, 17 [-1.82; 0.04]  GO_0005319_LIPID TRANSPORTER ACTIVITY: MF, 21 [-1.80; 0.04]  GO_0016787_HYDROLASE ACTIVITY: MF, 156 [-1.80; 0.03]  GO_0004565_BETA-GALACTOSIDASE ACTIVITY: MF, 14 [-1.80; 0.03]GO_0000786_NUCLEOSOME: CC, 16 [-1.78; 0.03] | N/A | GO_0042302_STRUCTURAL CONSTITUENT OF CUTICLE: MF, 68 [-2.11; 0.005]  GO_0034993_LINC COMPLEX: CC, 12 [-1.97; 0.02] |
| Conventional vs. Organic | # of enriched GO terms | 11 (0/11) | 18(1/17) | 2 (1/1) |
|  | Enriched # of connections | 0/10 (S2 iiia) | 0/19 (S2 iiib) | 0/0 (S2 iiic) |
|  | Positively enriched in G1, relative to G2: GO terms | N/A | GO_0004181_METALLOCARBOXYPEPTIDASE ACTIVITY: MF, 20 [1.87; 0.02] | GO_0034993_LINC COMPLEX: CC, 12 [2.02; 0.04] |
|  | Negatively enriched in G1, relative to G2: GO terms | GO_0005549_ODORANT BINDING: MF, 103 [-1.99; 0.002]  GO_0004313_[ACYL-CARRIER-PROTEIN] S-ACETYLTRANSFERASE ACTIVITY: MF, 25 [-1.85; 0.02]  GO_0004316_3-OXOACYL-[ACYL-CARRIER-PROTEIN] REDUCTASE (NADPH) ACTIVITY: MF, 25 [-1.84; 0.01]  GO_0006412_TRANSLATION: BP, 144 [-1.84; 0.01]  GO_0004319_ENOYL-[ACYL-CARRIER-PROTEIN] REDUCTASE (NADPH, B-SPECIFIC) ACTIVITY: MF, 25 [-1.83; 0.01]  GO_0016296_PALMITOYL-[ACYL-CARRIER-PROTEIN] HYDROLASE ACTIVITY: MF, 25 [-1.83; 0.01]  GO_0003735_STRUCTURAL CONSTITUENT OF RIBOSOME: MF, 141 [-1.83; 0.01]  GO_0004317_3-HYDROXYPALMITOYL-[ACYL-CARRIER-PROTEIN] DEHYDRATASE ACTIVITY: MF, 25 [-1.82; 0.01]  GO_0016295_MYRISTOYL-[ACYL-CARRIER-PROTEIN] HYDROLASE ACTIVITY: MF, 25 [-1.82; 0.01]  GO_0004320_OLEOYL-[ACYL-CARRIER-PROTEIN] HYDROLASE ACTIVITY: MF, 25 [-1.82; 0.009]  GO_0005840_RIBOSOME: CC, 153 [-1.80; 0.01] | GO_0016491_OXIDOREDUCTASE ACTIVITY: MF, 436 [-2.38; 0]  GO_0008234_CYSTEINE-TYPE PEPTIDASE ACTIVITY: MF, 116 [-2.36; 0]  GO_0050790_REGULATION OF CATALYTIC ACTIVITY: BP, 42 [-2.36; 0]  GO_0004197_CYSTEINE-TYPE ENDOPEPTIDASE ACTIVITY: MF, 53 [-2.34; 0]  GO_0016705_OXIDOREDUCTASE ACTIVITY, ACTING ON PAIRED DONORS, WITH INCORPORATION OR REDUCTION OF MOLECULAR OXYGEN: MF, 313 [-2.29; 0]  GO_0004497_MONOOXYGENASE ACTIVITY: MF, 314 [-2.26; 0]  GO_0016772_TRANSFERASE ACTIVITY, TRANSFERRING PHOSPHORUS-CONTAINING GROUPS: MF, 219 [-2.23; 0]  GO_0005975_CARBOHYDRATE METABOLIC PROCESS: BP, 424 [-2.23; 0]  GO_0008810_CELLULASE ACTIVITY: MF, 43 [-2.22; 0]  GO_0005506_IRON ION BINDING: MF, 374 [-2.19; 0]  GO_0020037_HEME BINDING: MF, 362 [-2.17; 0]  GO_0004252_SERINE-TYPE ENDOPEPTIDASE ACTIVITY: MF, 307 [-1.62; 0.03]  GO_0042626_ATPASE ACTIVITY, COUPLED TO TRANSMEMBRANE MOVEMENT OF SUBSTANCES: MF, 189 [-1.81; 0.002]  GO_0016758_TRANSFERASE ACTIVITY, TRANSFERRING HEXOSYL GROUPS: MF, 144 [-1.96; 1.63E-04]  GO_0030246_CARBOHYDRATE BINDING: MF, 145 [-2.06; 0]  GO_0004553_HYDROLASE ACTIVITY, HYDROLYZING O-GLYCOSYL COMPOUNDS: MF, 129 [-2.17; 0]  GO_0008234_CYSTEINE-TYPE PEPTIDASE ACTIVITY: MF, 116 [-2.36; 0] | GO_0030245_CELLULOSE CATABOLIC PROCESS: BP, 20 [-1.68; 0.048] |
| Switch vs. Maintain | # of enriched GO terms | 4 (4/0) | 25(15/10) | N/A |
|  | Enriched # of connections | 2/0 | 15/6 | N/A |
|  | Positively enriched in G1, relative to G2: GO terms | GO_0004650_POLYGALACTURONASE ACTIVITY: MF, 39 [1.97; 0]  GO_0030599_PECTINESTERASE ACTIVITY: MF, 30 [1.94; 0]  GO_0045330_ASPARTYL ESTERASE ACTIVITY: MF, 30 [1.90; 0]  GO_0044822_POLY(A) RNA BINDING: MF, 17 [1.62; 0.02] | GO_0004650_POLYGALACTURONASE ACTIVITY: MF, 39 [2.49; 0]  GO_0008234_CYSTEINE-TYPE PEPTIDASE ACTIVITY: MF, 116 [2.41; 0]  GO_0004197_CYSTEINE-TYPE ENDOPEPTIDASE ACTIVITY: MF, 53 [2.32; 0]GO_0005975_CARBOHYDRATE METABOLIC PROCESS: BP, 424 [2.31; 0]  GO_0050790_REGULATION OF CATALYTIC ACTIVITY: BP, 42 [2.29; 0]  GO_0030599_PECTINESTERASE ACTIVITY: MF, 30 [2.23; 0]  GO_0045330_ASPARTYL ESTERASE ACTIVITY: MF, 30 [2.21; 0]  GO_0016829_LYASE ACTIVITY: MF, 47 [2.15; 0]  GO_0008810_CELLULASE ACTIVITY: MF, 43 [2.09; 0]  GO_0016787_HYDROLASE ACTIVITY: MF, 323 [2.01; 0]  GO_0016491_OXIDOREDUCTASE ACTIVITY: MF, 436 [1.93; 1.50E-04]  GO_0005576_EXTRACELLULAR REGION: CC, 287 [1.65; 0.03]  GO_0030246_CARBOHYDRATE BINDING: MF, 145 [1.87; 7.85E-04]  GO_0008643_CARBOHYDRATE TRANSPORT: BP, 72 [1.61; 0.046]  GO_0022891_SUBSTRATE-SPECIFIC TRANSMEMBRANE TRANSPORTER ACTIVITY: MF, 69 [1.73; 0.01] | N/A |
|  | Negatively enriched in G1, relative to G2: GO terms | N/A | GO_0008061_CHITIN BINDING: MF, 87 [-2.24; 0]  GO_0004181_METALLOCARBOXYPEPTIDASE ACTIVITY: MF, 20 [-2.12; 7.32E-04]  GO_0006030_CHITIN METABOLIC PROCESS: BP, 77 [-2.10; 4.88E-04]  GO_0005319_LIPID TRANSPORTER ACTIVITY: MF, 38 [-1.94; 0.007]  GO_0003993_ACID PHOSPHATASE ACTIVITY: MF, 44 [-1.87; 0.02]  GO_0006357_REGULATION OF TRANSCRIPTION FROM RNA POLYMERASE II PROMOTER: BP, 22 [-1.85; 0.02]  GO_0045735_NUTRIENT RESERVOIR ACTIVITY: MF, 19 [-1.86; 0.03]  GO_0004177_AMINOPEPTIDASE ACTIVITY: MF, 65 [-1.82; 0.03]  GO_0004697_PROTEIN KINASE C ACTIVITY: MF, 17 [-1.80; 0.04]  GO_0008237_METALLOPEPTIDASE ACTIVITY: MF, 73 [-1.79; 0.04] | N/A |
