# Supplementary material for: Host-specific gene expression as a tool for introduction success in Naupactus parthenogenetic weevils
Source: PLoS One. 2021 Jul 30;16(7):e0248202. doi: 10.1371/journal.pone.0248202 (PMC8323892; doi:10.1371/journal.pone.0248202)
Supplement: S5 Table — Description of Trinity alignment parameters and R packages including the sources for each module. (DOCX) [file pone.0248202.s007.docx]

**S Table. Summary of computational resources used in this study.**  Description of Trinity alignment parameters and R packages, including the sources for each module.

| Transcriptome alignment step | Parameters |
| --- | --- |
| Trinity script align_and_estimate_abundance.pl | --seqType fq --SS_lib_type RF --output_dir abundance --thread_count 6 --left [file_R1] --right [file_R2] --output_prefix [sample] --est_method RSEM --aln_method bowtie --trinity_mode |
| Trinity --aln_method bowtie parameters | --all --best --strata -m 300 --chunkmbs 512 |
| Trinity --est_method RSEM parameters | rsem-calculate-expression default parameters (no options specified as paired-end alignment and trimming has already occurred) |

| **R software and packages** | **Citations and source** |
| --- | --- |
| gridExtra | Auguie B, Antonov A. gridExtra: Miscellaneous Functions for “Grid” Graphics [Internet]. 2017 [cited 2020 Mar 17]. Available from: [https://CRAN.R-project.org/package=gridExtra](https://cran.r-project.org/package=gridExtra) |
| VennDiagram | Chen H. VennDiagram: Generate High-Resolution Venn and Euler Plots [Internet]. 2018 [cited 2020 Mar 17]. Available from: [https://CRAN.R-project.org/package=VennDiagram](https://cran.r-project.org/package=VennDiagram)  Chen H, Boutros PC. VennDiagram: a package for the generation of highly-customizable Venn and Euler diagrams in R. BMC Bioinformatics [Internet]. 2011 Jan 26 [cited 2020 Jan 22];12(1):35. Available from:<https://doi.org/10.1186/1471-2105-12-35> |
| ggbeeswarm | Clarke E, Sherrill-Mix S. ggbeeswarm: Categorical Scatter (Violin Point) Plots [Internet]. 2017 [cited 2020 Mar 17]. Available from: [https://CRAN.R-project.org/package=ggbeeswarm](https://cran.r-project.org/package=ggbeeswarm) |
| data.table | Dowle M, Srinivasan A, Gorecki J, Chirico M, Stetsenko P, Short T, et al. data.table: Extension of “data.frame” [Internet]. 2019 [cited 2020 Mar 17]. Available from: [https://CRAN.R-project.org/package=data.table](https://cran.r-project.org/package=data.table) |
| Hmisc | Jr FEH, others with contributions from CD and many. Hmisc: Harrell Miscellaneous [Internet]. 2020 [cited 2020 Mar 17]. Available from: [https://CRAN.R-project.org/package=Hmisc](https://cran.r-project.org/package=Hmisc) |
| ggpubr | Kassambara A. ggpubr: “ggplot2” Based Publication Ready Plots [Internet]. 2020 [cited 2020 Mar 17]. Available from: [https://CRAN.R-project.org/package=ggpubr](https://cran.r-project.org/package=ggpubr) |
| RColorBrewer | Neuwirth E. RColorBrewer: ColorBrewer Palettes [Internet]. 2014 [cited 2020 Mar 17]. Available from: [https://CRAN.R-project.org/package=RColorBrewer](https://cran.r-project.org/package=RColorBrewer) |
| R base language | R Core Team. R: A language and environment for statistical computing. [Internet]. Vienna, Austria: R Foundation for Statistical Computing; 2019. Available from: [https://www.R-project.org/](https://www.r-project.org/) |
| gplots | Warnes GR, Bolker B, Bonebakker L, Gentleman R, Huber W, Liaw A, et al. gplots: Various R Programming Tools for Plotting Data [Internet]. 2020 [cited 2020 Mar 17]. Available from: [https://CRAN.R-project.org/package=gplots](https://cran.r-project.org/package=gplots) |
| reshape | Wickham H. Reshaping Data with the reshape Package. Journal of Statistical Software [Internet]. 2007 Nov 13 [cited 2020 Mar 17];21(1):1–20. Available from:<https://www.jstatsoft.org/index.php/jss/article/view/v021i12> |
| tidyverse | Wickham H, Averick M, Bryan J, Chang W, McGowan L, François R, et al. Welcome to the Tidyverse. Journal of Open Source Software [Internet]. 2019 Nov 21 [cited 2020 Mar 17];4(43):1686. Available from:<https://joss.theoj.org/papers/10.21105/joss.01686> |
| magrittr | Wickham SMB and H. magrittr: A Forward-Pipe Operator for R [Internet]. 2014 [cited 2020 Mar 17]. Available from: [https://CRAN.R-project.org/package=magrittr](https://cran.r-project.org/package=magrittr) |
